# Hyperlactatemia associated with diabetic ketoacidosis in pediatric intensive care unit

**DOI:** 10.1186/s12902-021-00776-9

**Published:** 2021-05-27

**Authors:** Jingwei Liu, Haibo Yan, Yumei Li

**Affiliations:** Department of Pediatric Intensive Care Unit, The First Hospital of Jilin University, Xin Min Street, 130021 Changchun, China

**Keywords:** Diabetic ketoacidosis, Diabetes mellitus, Lactate

## Abstract

**Background:**

Children with diabetic ketoacidosis often have elevated lactate. In this study, we investigated the clinical variables associated with hyperlactatemia in children with diabetic ketoacidosis.

**Methods:**

We designed a single-center retrospective descriptive study of children with diabetic ketoacidosis in a pediatric intensive care unit.

**Results:**

Of the 107 patients with diabetic ketoacidosis included in the analysis, 61 developed hyperlactatemia. Multivariate logistic regression analysis showed that heart rate (*p* = 0.003),diastolic blood pressure (*p* = 0.001) and stage of severity (*p* = 0.042) were independently associated with the development of hyperlactatemia in diabetic ketoacidosis. We found that lactate level was not significantly associated with length of hospital stay (*p* = 0.115) or the length of time to diabetic ketoacidosis resolution (*p* = 0.143).

**Conclusions:**

Children with diabetic ketoacidosis presenting with severer stage, elevated heart rate and higher diastolic blood pressure may be prone to hyperlactatemia. Hyperlactatemia was not associated with length of time to DKA resolution and length of hospital stay.

## Background

Diabetic ketoacidosis (DKA) is an acute complication of diabetes mellitus typically characterized by hyperglycemia, ketonemia, and acidemia [1]. DKA typically occurs in patients with established diabetes mellitus who underwent insulin omission or inappropriate management of intercurrent illness or in patients with new-onset diabetes [2]. The biochemical criteria for ketoacidosis are: (1)pH < 7.3, (2) serum bicarbonate < 15 mmol/L, (3)plasma glucose concentration > 11 mmol/L, and (4)ketonuria and presence of ketones in serum [3]. Ketoacidosis is divided into three stages of severity based on pH and bicarbonate levels [4]. Mild stage ketoacidosis is defined as pH < 7.3 or bicarbonate < 15 mmol/L, moderate stage is pH < 7.2 orbicarbonate < 10 mmol/L, severe stage is pH < 7.1 or bicarbonate <5 mmol/L [4].

Lactic acidosis is a common complication in children with DKA in the emergency department [5]. Lactate concentrations > 2mmol/L is a part of the definition of septic shock [6]. Elevated serum lactate levels are usually considered as a marker for hypoxia in tissues and used as a clinical indicator of sepsis severity and prognosis [7]. A retrospective observational study confirmed that hyperlactatemia can be an early indicator of organ failure [8]. Other studies have shown that serum lactate level can also be a predictor of mortality following burn injury [9] and an association between lactate clearance and in-hospital mortality in patients with cardiac arrest [10]. However, lactic acidosis has also been reported not to be associated with length of stay in the intensive care unit (ICU) or mortality in patients with DKA [11]. Lactic acidosis is defined as a lactate concentration ≥ 2.5 mmol/L [5], with severe lactic acidosis defined as blood lactate concentration > 5 mmol/L and anion gap ≥ 10 [12]. Hyperlactatemia is defined as lactate levels > 2 mmol/Lin a study about DKA [13]. Some studies about DKA and lactate showed that thiamine were inversely related to lactate [14], glucose was positively correlated with lactate [11]. Though resolution of DKA is often rapid, high lactate can persist [13]. This study aims to find predictors of hyperlactatemia and investigate the incidence and significance of hyperlactatemia in patients with DKA.

## Methods

We performed a retrospective study of patients with DKA who were admitted to the pediatric ICU in the First Hospital of Jilin University. Our department with more than 20 beds is the earliest and largest pediatric ICU in Jilin Province China and nearly 1000 patients are admitted every year. Almost all the emergency patients with DKA were admitted to our department. The protocol for the study project has been approved by the ethics committee of The First Hospital of Jilin University (2020 − 396) conforms to the provisions of the Declaration of Helsinki. The requirement for informed consent was abandoned in accordance with the rules of the ethics committee.

Patients were identified by electronic queries from the pediatric ICU medical records. Children with DKA admitted to pediatric ICU of the First Hospital of Jilin University between January 2017 and June 2020 were collected for analysis. The exclusion criteria were death or discharge within 24 h. Two trained researchers extracted the data from complete medical records. Age, sex, family history, clinical history, length of hospital stay, the length of time to resolution of DKA, laboratory examination results, and vital signs upon admission to pediatric ICU were recorded. The laboratory examination results included pH, leukocyte count, C reactive protein, procalcitonin, creatinine, blood urea nitrogen, alanine transaminase, triglyceride, ferritin, blood glucose, C peptide, glycosylated hemoglobin, and lactic dehydrogenase. All the blood samples were collected within 24 h after admission. The diagnosis of hyperlactatemia was based on the highest lactate level before the correction of ketoacidosis during hospitalization. All the patients with DKA received intravenous fluids and insulin to correct dehydration, electrolyte imbalances,reduce glucose levels and suppress ketogenesis after admission. After ketoacidosis was corrected, patients were transferred to pediatric endocrinology department for better monitoring blood glucose and adjusting insulin dose. Resolution of DKA was defined as pH > 7.3, bicarbonate > 18 mmol/L and urine ketones (1 + or 0).

### Statistical analysis

Data were recorded in a standardized electronic data collection table. Patients were excluded if they had DKA combined with other genetic metabolic diseases which are prone to develop hyperlactatemia, such as methylmalonic acidemia. Hyperlactatemia was defined as lactate levels > 2 mmol/L, which is consistent with a previous study [13]. All eligible patients with DKA were stratified into two groups according to lactate levels. Patients with lactate level > 2 mmol/L were in the hyperlactatemia group and patients with lactate level ≤ 2 mmol/L were in the non-hyperlactatemia group. The clinical data of these two groups were compared.

The data were described according to their distribution and type. Normally distributed data were represented as mean (SD), non-normally distributed data as median (lower quartiles, upper quartiles), and categorical data as proportions. Chi-square tests were used to compare categorical variables between the hyperlactatemia and non-hyperlactatemia low lactate level groups. Group comparisons for normally distributed variables were performed using a t-test, and the rank sum test was used for non-normally distributed variables. Multivariate logistic regression performed for independent variables were shown to be significant through univariate analysis. Spearman correlation coefficients were used to evaluate the univariate relationship between non-normally distributed continuous variables. Data analysis was performed using SPSS software version 23, with the level of significance set at *p* < 0.05.

## Results

There were 107 patients with DKA admitted to pediatric ICU from January 2017 to June 2020. None of these patients met the exclusion criteria set by this study. Of the 107 patients with DKA, 61 patients developed hyperlactatemia. The clinical characteristics of the patients in this study are presented in Table 1. There were several significant differences between clinical characteristics of the patients in the two groups. Patients in the hyperlactatemia group were significantly older and had faster heart rate, higher diastolic blood pressure,and lower levels of pH and C peptide. The patients with new onset diabetes mellitus in hyperlactatemia group was significantly less than that in non-hyperlactatemia group (*p* = 0.016). The severity of the two groups was different (*p* = 0.044). They also had higher leukocyte counts, procalcitonin, blood glucose, creatinine, and blood urea nitrogen than that of the non-hyperlactatemia group (Table 1). Multivariate logistic regression analysis showed that, both heart rate(*p* = 0.003),diastolic blood pressure (*p* = 0.001) and stage of severity (*p* = 0.042)were independently associated with the development of hyperlactatemia in DKA (Table 2).

**Table 1:** Patient clinical manifestation stratified according to lactate level

Variables	Lactate > 2mmol/L (*n* = 61)	Lactate ≤ 2mmol/L (*n* = 46)	*p*	*z/t/χ*^*2*^
Age (years)	11(8.500, 14)	8(4, 11)	< 0.001	-3.625
Female (%)	38(62.295 %)	27(58.696 %)	0.706	0.142
infection	28(45.902 %)	23(50 %)	0.674	0.177
new onset diabetes mellitus (%)	39(63.934 %)	39(84.783 %)	0.016	5.769
Family history of diabetes mellitus (%)	6(9.836 %)	9(19.565 %)	0.151	2.059
Heart rate (beats/minute)	133.508(5.375)	120.543(19.873)	< 0.001	3.806
Systolic blood pressure(mmHg)	114.183(20.340)	110.565(17.735)	0.340	0.959
Diastolic blood pressure(mmHg)	73(56.500, 81)	66(57.75, 74)	0.028	-2.191
PH	7.025(0.148)	7.098(0.167)	0.020	2.368
Leukocyte count(10^9^/L)	18.41(12.990, 32.285)	13.295(7.978, 23.463)	0.004	-2.898
C reactive protein(mg/L)	8.13(4.435, 26.570)	4.765(1.775, 11.955)	0.167	-1.381
Procalcitonin (ng/mL)	1.11(0.235, 8.058)	0.13(0.085, 1.455)	< 0.001	-3.744
Creatinine (umol/L)	44(34.625, 64.300)	30.1(23.875, 38.625)	< 0.001	-4.153
Blood urea nitrogen (mmol/L)	5.905(3.473, 10.388)	4.005(2.370, 5.653)	0.002	-3.037
Alanine transaminase (U/L)	13.3(9.650, 19.75)	11.1(8.425, 15.65)	0.107	-1.611
Triglycerides (mmol/L)	1.82(1.185, 2.968)	1.69(1.045, 3.32)	0.336	-0.962
Ferritin (ug/L)	180.8(120.950, 322.725)	153(83.825, 222.975)	0.105	-1.622
Blood glucose (mmol/L)	27.8(19.050, 29)	20.85(16.35, 27.35)	0.002	-3.05
C peptide (nmol/L)	0.060(0.010, 0.155)	0.135(0.09, 0.23)	0.003	-2.99
Glycosylated hemoglobin (%)	12.485(2.591)	12.917(2.347)	0.377	-0.887
Lactic dehydrogenase (U/L)	289.5(217.75, 392.75)	258(209, 336)	0.144	-1.46
**Stage of severity**			0.044	6.246
Mild stage	2	8		
Moderate stage	14	10		
Severe stage	45	28		

**Table 2:** Multivariate logistic regression analysis of factors associated with hyperlactemia

Variables	B	S.E	Wald	*p*	OR	95 % CI	
						Lower	Upper
Heart rate	0.085	0.027	10.087	0.001	1.089	1.033	1.148
Diastolic blood pressure	0.087	0.028	9.561	0.002	1.091	1.032	1.153
Leukocyte count	0.007	0.031	0.046	0.829	1.007	0.947	1.071
Creatinine	0.038	0.030	1.642	0.200	1.039	0.980	1.102
Procalcitonin	0.003	0.048	0.004	0.949	1.003	0.914	1.101
Age	0.106	0.102	1.089	0.297	1.112	0.911	1.358
Blood urea nitrogen	0.038	0.075	0.252	0.616	1.038	0.896	1.203
C peptide	-3.563	1.827	3.804	0.051	0.028	0.001	1.018
New onset diabetes mellitus	1.281	0.859	2.222	0.136	3.600	0.668	19.401
Blood glucose	0.084	0.050	2.814	0.093	1.087	0.986	1.199
Stage of severity							
Mild VS Severe	-0.758	1.234	0.377	0.539	0.468	0.042	5.263
Moderate VS Severe	2.483	1.106	5.038	0.025	11.978	1.370	104.717

**Fig. 1:**
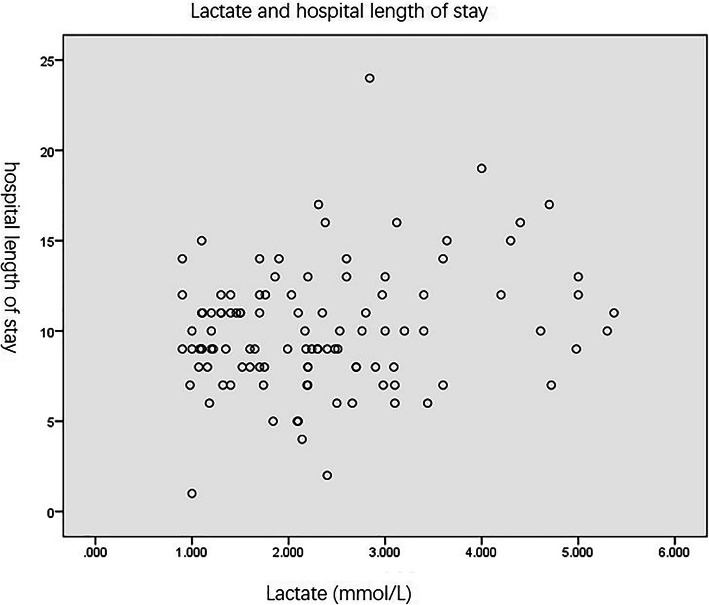
Lactate levels and hospital length of stay. In patients with DKA, lactate was not associated with hospital length of stay (*r* = 0.154, *p* = 0.115)

**Fig. 2:**
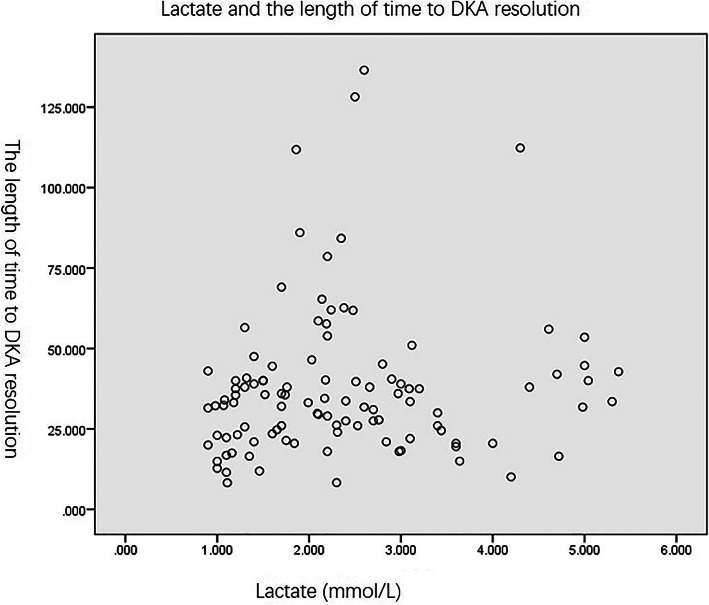
Lactate levels and the length of time to resolution of DKA. In patients with DKA, lactate was not associated with the length of time to resolution of DKA (*r* = 0.143, *p* = 0.143)

Spearman correlation was used to determine the association between the length of hospital stay and lactate levels. The length of hospital stay in our study included the total length of stay in the department of pediatric endocrinology and pediatric ICU. The average length of hospital stay was 9(8, 11)days in the non-hyperlactatemia group and 10(8, 12.75) days in the hyperlactatemia group (*p* = 0.722). The *r* value between length of stay and lactate levels indicated a very low correlation of *r* = 0.154 with an insignificant p-value of 0.115 (Fig. 1). The Spearman correlation was also used to calculate the correlation between length of time to DKA resolution and lactate levels. The average length of time to DKA resolution was 32.3(21.21,39.5) hours in the non-hyperlactatemia group and 34.5(26, 48.75) hours in the hyperlactatemia group (*p* = 0.098). Our results showed a very low correlation of *r* = 0.143 between length of time to DKA resolution and lactate levels, with an insignificant *p*-value of 0.143 (Fig. 2).

## Discussion

This study was a large single-center retrospective study of hyperlactatemia in patients with DKA. We assessed the incidence, characteristics, and clinical outcomes of patients with hyperlactatemia induced by DKA and identified independent risk factors for the development of hyperlactatemia.

The clinical manifestations of DKA include dehydration, Kussmaul respiration, nausea, vomiting, abdominal pain, loss of consciousness, leukocytosis, and increased serum amylase [4].A study showed that mortality rate was 9 %, and readmission for DKA rate was 31 % during over 7 years of follow-up of patients with DKA [15]. DKA mainly occurs in patients with type 1 diabetes mellitus, but it can also occur in patients with type 2 diabetes mellitus [16]. Hyperlactatemia which is defined as lactate > 2mmol/L is common in patients with DKA [11, 13]. Metabolically, hyperlactatemia occurs when lactate production is greater than lactate consumption. In general, hyperlactatemia is caused by both disorders associated with tissue hypoxia and disorders unassociated with tissue hypoxia [17]. Possible pathogenic mechanisms may include anaerobic glycolysis in the hypoperfusion region, decreased lactate clearance of liver, stress-related adrenergic-induced aerobic glycolysis, and mitochondrial dysfunction [18–21]. In our study, 57 % of patients with DKA developed hyperlactatemia. This prevalence is lower than those reported in the previous studies 90 % [13], 67.9 % [22], and 68 % [11].

The mechanism by which patients with DKA develop hyperlactatemia has not been clearly described yet. There were more patients with new-onset diabetes mellitus in our hyperlactatemia group than in the non-hyperlactatemia group. Children whose parents were unaware of the symptoms of diabetes mellitus may be more prone to DKA during their initial diagnosis of diabetes mellitus [23]. Another study showed that a low education level of parents was reflected in an increased severity of DKA [24]. Both leukocyte count and procalcitonin levels were higher in the patients with hyperlactatemia than in those without. Patients with DKA may develop non-infectious systemic inflammation exemplified by increased leukocyte count [25] and procalcitonin [26, 27]. Non-infectious systemic inflammation is different from severe infectious diseases, which can easily lead to elevated lactic acid and is quickly relieved after DKA treatment. Prior research has found that lactic acidosis was significantly associated with a higher blood glucose level [5, 11]. Similarly, our results found that blood glucose in patients with hyperlactatemia was significantly higher than in patients without. It is noteworthy that C peptide was also significantly lower in patients with hyperlactatemia, which may explain the significantly higher blood sugar levels in them.

Our results found that patients with hyperlactatemia had higher creatinine and blood urea nitrogen. The levels of creatinine and blood urea nitrogen could not meet the criteria for diagnosis of acute kidney injury. A previous study showed that acute kidney injury occurred in nearly 30 % of patients with diabetes [28]. The renal cortex plays an important role in the clearance of lactate [29]. However,we found that higher creatinine and urea nitrogen was not an independent risk factor for hyperlactatemia in DKA patients. This may be because acidosis could increase the ability of the kidneys to clear lactate [30].

A previous study indicated that the maximal lactate level 24 h after ICU admission was strongly associated with in-hospital mortality and 90-day survival in patients [31]. Another study also showed that serum lactate can be a predictor of mortality in emergency department patients with infection [32]. These studies all involved mortality, but there were no deaths in our study, so we studied the correlation between lactate and length of hospital stay and time till resolution of DKA. Another study showed a negative correlation between lactate levels and DKA recovery time [22]. However, we found that lactate levels were not associated with the length of hospital stay or time till resolution of DKA. Our results suggest that lactate level should not be used as a predictor of clinical outcomes in DKA. Furthermore, a previous study on adult diabetic ketoacidosis also showed that lactic acidosis could not predict the clinical prognosis of patients with DKA [11].

Our multivariate logistic regression analysis showed that heart rate ,diastolic blood pressure and stage of severity were independently associated with a higher risk of hyperlactatemia in DKA. DKA is often accompanied by dehydration [33]. Patients with DKA may develop hypovolemia due to fluid loss [34]. Hypovolemia may be a cause for cerebral edema in DKA [35]. Despite dehydration, hypertension may still occur in some children with DKA[36]. Paradoxical hypertension during DKA might be the result of reflexive regulatory responses that maintain normal cerebral perfusion pressure during cerebral edema [34]. Renal injury might also play a role in the development of hypertension in patients with DKA [34]. Patients with hypovolemia can manifest diminished internal diameter of the aorta, inferior vena cava, superior mesenteric vein and artery [37]. The contraction of blood vessels can maintain normal blood pressure or elevate blood pressure. These changes in blood pressure and heart rate are responses to hypovolemia associated with diabetic ketosis. Therefore, we propose that hypovolemia may be the cause of hyperlactatemia in diabetic ketosis.

There are several limitations to our investigation. The data were obtained retrospectively from medical records, whose accuracy depends on appropriate diagnostic record-keeping. Of the patients included in this study, only one had died. This low incidence of mortality limits the power to detect the difference in the outcome of variables between the two groups in our study. Additionally, this study was only representative of single-center pediatric ICU and the sample size was relatively small. In the future, it is necessary to further study the prevalence, pathogenesis, and clinical significance of hyperlactatemia in DKA patients.

## Conclusions

Hyperlactatemia is common in patients with DKA. Overall, our results suggest that diastolic blood pressure, heart rate might and stage of severity may be predictors for hyperlactatemia in DKA. We further found that hyperlactatemia was not a prognostic marker of DKA and hyperlactatemia was not associated with length of time to DKA resolution and length of hospital stay.

## Data Availability

The dataset supporting the conclusions of this article is included within the article.

## Abbreviations

DKA: Diabetic ketoacidosis
ICU: Intensive care unit
